# Profiling Distinctive Inflammatory and Redox Responses to Hydrogen Sulfide in Stretched and Stimulated Lung Cells

**DOI:** 10.3390/antiox11051001

**Published:** 2022-05-19

**Authors:** Sashko G. Spassov, Simone Faller, Andreas Goeft, Marc-Nicolas A. von Itter, Andreas Birkigt, Peter Meyerhoefer, Andreas Ihle, Raphael Seiler, Stefan Schumann, Alexander Hoetzel

**Affiliations:** Department of Anesthesiology and Critical Care, Medical Center—University of Freiburg, Faculty of Medicine, University of Freiburg, Hugstetter Str. 55, 79106 Freiburg, Germany; simone.faller@uniklinik-freiburg.de (S.F.); andreas.goeft@gmx.de (A.G.); marc-nicolas.itter@uniklinik-freiburg.de (M.-N.A.v.I.); andreas.birkigt@uniklinik-freiburg.de (A.B.); petermeyerhoefer@web.de (P.M.); andi-ihle@gmx.de (A.I.); raphaelseiler85@me.com (R.S.); stefan.schumann@uniklinik-freiburg.de (S.S.); alexander.hoetzel@uniklinik-freiburg.de (A.H.)

**Keywords:** hydrogen sulfide, cell-type specific response, oxidative stress, inflammatory signaling, redox homeostasis, epithelial, endothelial, macrophage, neutrophil cells

## Abstract

Hydrogen sulfide (H_2_S) protects against stretch-induced lung injury. However, the impact of H_2_S on individual cells or their crosstalk upon stretch remains unclear. Therefore, we addressed this issue in vitro using relevant lung cells. We have explored (i) the anti-inflammatory properties of H_2_S on epithelial (A549 and BEAS-2B), macrophage (RAW264.7) and endothelial (HUVEC) cells subjected to cycling mechanical stretch; (ii) the intercellular transduction of inflammation by co-culturing epithelial cells and macrophages (A549 and RAW264.7); (iii) the effect of H_2_S on neutrophils (Hoxb8) in transmigration (co-culture setup with HUVECs) and chemotaxis experiments. In stretched epithelial cells (A549, BEAS-2B), the release of interleukin-8 was not prevented by H_2_S treatment. However, H_2_S reduced macrophage inflammatory protein-2 (MIP-2) release from unstretched macrophages (RAW264.7) co-cultured with stretched epithelial cells. In stretched macrophages, H_2_S prevented MIP-2 release by limiting nicotinamide adenine dinucleotide phosphate oxidase-derived superoxide radicals (ROS). In endothelial cells (HUVEC), H_2_S inhibited interleukin-8 release and preserved endothelial integrity. In neutrophils (Hoxb8), H_2_S limited MIP-2-induced transmigration through endothelial monolayers, ROS formation and their chemotactic movement. H_2_S induces anti-inflammatory effects in a cell-type specific manner. H_2_S limits stretch- and/or paracrine-induced inflammatory response in endothelial, macrophage, and neutrophil cells by maintaining redox homeostasis as underlying mechanism.

## 1. Introduction

A frequent complication of mechanical ventilation is the development of ventilator-induced lung injury (VILI) in patients with or without pre-existing pulmonary diseases [1,2]. Despite the optimization of ventilator settings in recent years, VILI and the acute respiratory distress syndrome still contribute to high rates of mortality and morbidity [3], as obviously seen these days during the COVID-19 pandemic [4].

VILI arises from repetitive unphysiological stretching of the lung and is characterized by tissue disruption, pulmonary edema, sequestration of immunocompetent cells, and release of reactive oxygen species [1] and pro-inflammatory cytokines [5,6,7]. Among all pro-inflammatory cytokines, interleukin-8 (IL-8) and its murine functional homologue, macrophage inflammatory protein-2 (MIP-2), have been demonstrated to participate in the development of VILI. Furthermore, IL-8/MIP-2 is able to directly recruit immunocompetent cells into target-tissues, e.g., the lung [8,9]. As a marker for lung injury, IL-8/MIP-2 are established for various cell types as a reliable indicator of cellular damage in vivo and in vitro [10,11]. In this respect, the early activation of pro-inflammatory signaling (including IL-8/MIP-2) in epithelial cells and resident alveolar macrophages may lead to epithelial and endothelial barrier dysfunction, and subsequently to influx and activation of neutrophils [5,12]. Activated neutrophils, in turn, exacerbate lung injury [13].

Hydrogen sulfide (H_2_S) or H_2_S-releasing salts (e.g., sodium hydrosulfide, sodium sulfide) have been shown to prevent both the release of pro-inflammatory cytokines and ROS in lung tissue, thus protecting against VILI in vivo [14,15,16,17]. Despite increasing evidence on the protective properties of H_2_S, little is known about the effects of H_2_S on cell-specific responses, particularly concerning intercellular crosstalk and interactions in propagation of inflammation during stretch. 

Therefore, the aim of the current study was to determine the role of H_2_S at the onset and propagation of inflammation, focusing on redox homeostasis. We investigated the effects of stretch and H_2_S on epithelial (A549, BEAS-2B) and macrophage (RAW264.7) cells as early mediators of inflammation in single cell and co-culture experimental setups. Moreover, we examined the role of stretch and H_2_S on endothelial (HUVEC) cells. Because endothelial cell layers build a barrier within the lungs, tissue disruption due to unphysiological stretch promotes neutrophil transmigration [13], thus propagating inflammation. Neutrophil influx through endothelial layers in turn is a key event in lung injury development [13,18]; therefore, it was necessary to analyze this in the co-culture setup. Moreover, we explored the direct impact of H_2_S on neutrophil (Hoxb8) transmigration and chemotaxis.

## 2. Materials and Methods

All chemical compounds, unless otherwise indicated, were purchased from Sigma-Aldrich (Taufkirchen, Germany).

### 2.1. Cell Cultures

Human A549-type II pneumocyte and murine RAW264.7 macrophage cell lines were obtained from the German Collection of Microorganisms and Cell Cultures (Braunschweig, Germany); human BEAS-2B bronchial epithelial cell line was obtained from Sigma-Aldrich (Taufkirchen, Germany) and primary human umbilical vein endothelial cells (HUVEC) from Pelobiotech (Planegg, Germany). Murine hematopoietic progenitor Hoxb8 neutrophils [19] were a generous gift from Prof. Georg Häcker (Institute of Medical Microbiology and Hygiene, UMC, Freiburg, Germany).

Cell cultures were grown under standard conditions using corresponding culture medium containing 1% penicillin-streptomycin (100 U/mL) as follows: A549 and RAW264.7 in DMEM-GlutaMAX supplemented with 10% fetal bovine serum (FBS; Gibco, Life Technologies, Paisley, Scotland), BEAS-2B in RPMI 1640-GlutaMAX (Gibco) supplemented with 5% FBS, primary HUVEC cells in Endothelial Cell Growth Medium Kit (PromoCell, Heidelberg, Germany), and Hoxb8 neutrophils in Opti-MEM-GlutaMAX (Gibco) supplemented with 10% heat-inactivated FBS, 30 µM 2-mercaptoethanol (Gibco), 1 µM estradiol and 1% stem cell factor. Prior to the experiments, progenitor Hoxb8 neutrophils were cultured for four days in differentiating medium (estradiol free medium), as described previously [19].

### 2.2. Cell Stretch Experiments

Cyclic stretch was applied using a Flexcell FX-5000 tension system (Flexcell International Corporation, Burlington, NC, USA). Specific cyclic stretch settings, exposure time, culture plates, medium and H_2_S releasing compounds were applied to each particular cell type at a frequency of 12 cycles/min, as shown in Table 1.

We used the fast H_2_S-releasing salt sodium hydrosulfide (NaHS) for short term (up to 6 h for A549, BEAS-2B, RAW264.7, and Hoxb8 cells) or the slow H_2_S-releasing compound morpholin-4-ium 4-methoxyphenyl (morpholino) phosphinodithioate dichloromethane complex (GYY4137) for long-term experiments based on the H_2_S-releasing kinetics of these two compounds [20] and the need to provide sufficient H_2_S for the 24 h experiments with the HUVEC cells. Dose response experiments were performed for each cell line, as representatively depicted in Supplementary Figure S1 for A549, or were based on previous reports [21].

Cells were seeded on collagen I or IV coated BioFlex culture plates (Flexcell International Corporation, Burlington, NC, USA) and grown to confluence. At the beginning of the experiments, growing medium was replaced by fresh medium and either left as control (control), incubated in the presence of NaHS (control + NaHS) or GYY4137 (control + GYY), or subjected to cyclic stretch in the absence (stretch) or presence of the respective H_2_S-releasing compound (stretch+NaHS/stretch + GYY4137).

In separate experiments, RAW264.7 macrophages were treated with the nicotinamide adenine dinucleotide phosphate (NADPH) oxidase inhibitor apocynin, dissolved in dimethylsulfoxide (DMSO). Cells were either incubated in cell culture medium supplemented with the DMSO (control) or with 2 mM apocynin (control + apocynin), or subjected to cyclic stretch in the absence (stretch) or presence of 2 mM apocynin (stretch + apocynin).

### 2.3. Co-Culture Experiments

**Figure 1:**
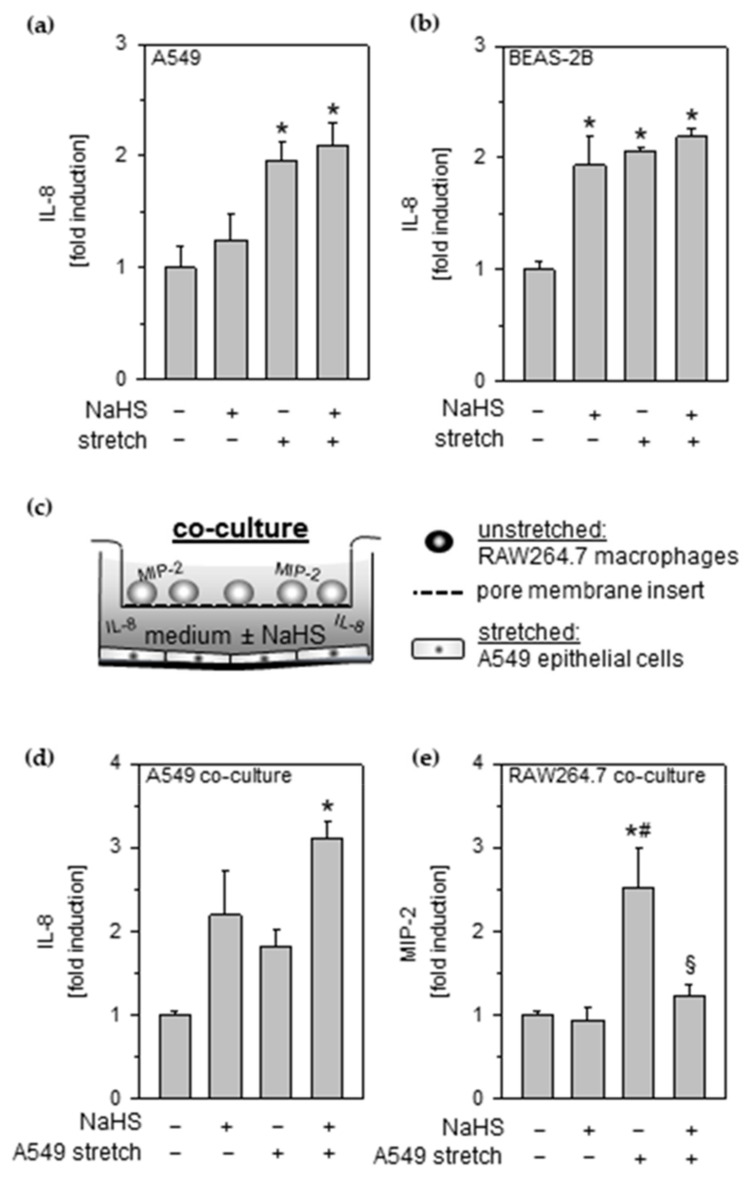
Effects of cyclic stretch and hydrogen sulfide on lung epithelial cells and on inflammatory signaling between epithelial cells and macrophages. Lung (**a**) A549 or (**b**) BEAS-2B epithelial cells were stretched and incubated with 1 mM NaHS, as indicated. The amounts of IL-8 in cell medium supernatant were determined by ELISA. (**c**) Setup for co-culture experiments. (**d**) A549 epithelial cells were stretched in co-culture and incubated with 1 mM NaHS, as indicated, and (**e**) RAW264.7 macrophages were grown in cell culture inserts with granted access to A549 cell culture medium. (**d**) The amounts of IL-8 released from human A549 and (**e**) MIP-2 released from murine RAW264.7 in cell medium supernatant were determined by ELISA. Data represent means ± SEM for n = 3/group. Analysis of variance (Tukey’s post hoc test), * *p* < 0.05 vs. control; # *p* < 0.05 vs. control + NaHS; § *p* < 0.05 vs. stretch.

A549 epithelial cells were seeded on the BioFlex plates. Separately, RAW264.7 macrophages were seeded and grown in cell-culture inserts (pore size 1.0 µm). At the beginning of the experiments, RAW264.7 macrophage inserts were transferred into the BioFlex plates on a spacer ring to prevent physical contact between the insert and the A549 monolayer (Figure 1c). A549 epithelial cells in the co-culture setup were either left unstretched (control/control + 1 mM NaHS) or were subjected to cyclic stretch (stretch/stretch + 1 mM NaHS) for 4 h.

### 2.4. Cytokine Measurements

The release of interleukin-8 (IL-8) and macrophage inflammatory protein-2 (MIP-2) were measured in cell culture supernatants using human IL-8 (Enzo Life Sciences, Lörrach, Germany) or murine CXCL2/MIP-2 Quantikine (R&D Systems, Wiesbaden, Germany) ELISA kits, respectively, according to the manufacturers’ instructions. 

### 2.5. Detection of Reactive Oxygen Species

In RAW264.7 macrophages and HUVEC endothelial cells, superoxide radical formation was detected using dihydroethidium (DHE, Thermo Fisher Scientific, Darmstadt, Germany) as described [22]. Mean fluorescence intensity was documented using laser- scanning microscopy and measured with ZEN 2 (blue edition 2011) software (Carl Zeiss, Jena, Germany).

ROS production in differentiated Hoxb8 neutrophils was induced by 10 µM phorbol 12-myristate 13-acetate (PMA) and detected by pre-incubation (15 min at 37 °C) with 5 µM 2′,7′-dichlorofluorescin diacetate (DCHF-DA) reagent. Live-dead cell staining was achieved using 3.74 µM propidium iodide (PI; Thermo Fisher Scientific, Darmstadt, Germany). Fluorescence intensity due to either PI or DCHF-DA staining was detected by flow cytometry (Attune Nxt, Thermo Fisher Scientific, Darmstadt, Germany). Mean fluorescence intensity was assessed and compared among the different groups using FlowJo v.X software (FlowJo LLC., Ashland, OR, USA).

### 2.6. Detection of Glutathione

Glutathione content in RAW264.7 macrophages was measured using ThiolTracker Violet (Thermo Fisher Scientific, Darmstadt, Germany); the staining was performed at the end of the experiments according to the manufacturers’ instructions. Mean fluorescence intensity was detected using laser-scanning microscopy and was measured with the ZEN software as described [23]. 

### 2.7. Detection of Mitochondrial Activity

In HUVEC endothelial cells, mitochondrial membrane potential was detected using MitoTracker Red dye (Molecular Probes, Thermo Fisher Scientific, Darmstadt, Germany), according to the manufacturer’s instructions. Mean fluorescence intensity was documented using Axio Imager Z.1 (Carl Zeiss, Jena, Germany) and measured with the ZEN software.

### 2.8. Actin Fluorescence Staining

Cytoskeleton of HUVEC endothelial cells was stained using Alexa Fluor 488 Phalloidin (Life Technologies). Fluorescent signals were documented with Axio Observer Z.1 microscope (Carl Zeiss). The percentage of cell-free areas was evaluated using ImageJ 1.47v (NIH, Bethesda, MD, USA) software [24].

### 2.9. Neutrophil Transmigration Assay

HUVEC endothelial cells were seeded onto cell culture inserts (pore size 3.0 µm). Prior to the experiments, cells were transferred to a new companion plate. Here, each well was divided into two compartments. The lower compartment was filled with 3 mL of neutrophil cell culture media, the upper compartment (containing the confluent HUVEC monolayer) with 1 mL of 5 × 10^6^ Hoxb8 neutrophil cell suspension. HUVEC and Hoxb8 neutrophil co-cultures were either left untreated (control) or were exposed to medium supplemented with 1 mM NaHS (control + NaHS). Transmigration was triggered by adding 1.33 pg/mL MIP-2 to the lower compartment without NaHS (MIP-2) or with 1 mM NaHS to the upper compartment (MIP-2 + NaHS). 

Furthermore, a series of experiments was performed adding 2 mM of the radical scavenger N-acetyl-L-cysteine (NAC) instead of NaHS, resulting in two experimental groups, i.e., control + NAC and MIP-2 + NAC.

After 2 h of incubation, inserts were removed from the companion plates. Living and dead cells from the lower and upper compartment were stained with PI, detected by flow cytometry (Attune Nxt, Thermo Fisher Scientific, Darmstadt, Germany) and analyzed using FlowJo software, as described above. The relative amount of migrated neutrophils was depicted as the ratio between living cells in the lower compartment and the total number of living cells.

### 2.10. Neutrophil Chemotaxis Assay

Neutrophil migration was assessed in chemotaxis µ-slides (ibidi GmbH, Martinsried, Germany). Hoxb8 cell suspension (10^6^ mL) was inserted in the observation channel. The surrounding chambers received estradiol free growing medium (control) or medium containing 1 mM NaHS (control + NaHS). A chemotaxis gradient was created by adding 1.3 ng/mL recombinant mouse MIP-2 into one of the µ-slide chambers either filled with 65 µL medium (MIP-2) or with medium containing 1 mM NaHS (MIP-2 + NaHS), according to the manufacturer’s instructions. Twenty minutes prior to time-lapse microscopy, µ-slides were incubated at 37 °C, 5% CO_2_ and sufficient humidity in a stage top incubator (Tokai Hit, Fujinomiya, Japan). Neutrophil migration was documented with an Axio Observer Z.1 inverted microscope equipped with a motorized xy-stage (Prior Scientific, Rockland, MA, USA). Images were captured every 2.5 min for 4 h using an EC Plan-Neofluar 5×/0.16 lens, Axiocam MRm camera and ZEN software. Cell tracking was performed in 97 consecutive images with ImageJ software using the manual tracking plug-in (Cordeli, F. Institut Curie, Orsay, France). Between 19 and 36 moving cells in each group were tracked per experiment. Chemotaxis characteristics such as velocity, displacement of the center of mass (average end position of tracked cells) and directness were evaluated using a ‘Chemotaxis and Migration Tool’, as described [25]. 

### 2.11. Statistical Analysis

The experiments were performed with samples from at least three subsequent cell passages (n = 3/group). Graphs were created with SigmaPlot 11.0 software (Systat Software Inc., Erkrath, Germany) and represent means ± standard error of means (SEM). Data were tested for normal variation and subsequently analyzed by one-way analysis of variance (ANOVA) for multiple-group comparison, followed by Tukey’s post hoc test using GraphPad Prism 7.01 (GraphPad Software, Inc., La Jolla, CA, USA). *p* < 0.05 was considered significant. 

Representative, rainbow false-colored images coding each pixel of a greyscale image with a distinct color depending on the pixel intensity were created with ZEN software for superoxide radical, glutathione and mitochondrial activity stainings.

‘Directness’ as a parameter for neutrophil cell migration is defined as the quotient of the net distance and the total distance the cells moved during the experiment. The more this value approaches 1, the more directional movement takes place. The ‘Rayleigh test’ is a statistical test to determine the distribution of all endpoints of the link ends, i.e., the cells, and their uniform, circular distribution around a given point (here the midpoint). With *p* < 0.05, the null hypothesis (i.e., the uniform, even distribution around the center) is rejected, and this can be interpreted as directed distribution or cell migration in our case. These calculated *p*-values were depicted in the corresponding graph. *p* < 0.05 was considered as directed cell migration [25].

## 3. Results

### 3.1. Effects of Cyclic Stretch and Hydrogen Sulfide on Lung Epithelial Cells

Epithelial cells are involved in pro-inflammatory response to mechanical stress. Compared to unstretched controls, incubation with NaHS slightly elevated IL-8 release from A549 epithelial cells (Figure 1a) while profoundly increasing IL-8 release from control BEAS-2B (Figure 1b). Upon cyclic stretch, IL-8 release was clearly augmented in both epithelial cell lines, irrespective of the absence or presence of NaHS (Figure 1a,b).

### 3.2. Effects of Cyclic Stretch and Hydrogen Sulfide on Inflammatory Signaling between Epithelial Cells and Macrophages

Next, we studied the effects of NaHS on the intercellular crosstalk between control or stretched A549 epithelial cells and unstretched RAW264.7 macrophages in a co-culture setup (Figure 1c). 

Application of NaHS as well as stretch or their combination resulted in an increased IL-8 release in stretched A549 epithelial cells (Figure 1d).

Focusing on MIP-2 release from co-cultured macrophages, we observed no response to NaHS incubation of unstretched epithelial cells (Figure 1e). However, macrophages significantly increased their MIP-2 release upon A549 stretching, indicating that an inflammatory signal was transmitted from stretched epithelial cells to unstreched macrophages. In sharp contrast, MIP-2 release was completely abolished in the presence of NaHS (Figure 1e).

### 3.3. Effects of Cyclic Stretch and Hydrogen Sulfide on Macrophages

Given the result that H_2_S modulated the crosstalk between stretched epithelial and unstretched macrophage cells, we next aimed to determine whether or not H_2_S acts as an anti-inflammatory on stretched macrophages.

**Figure 2:**
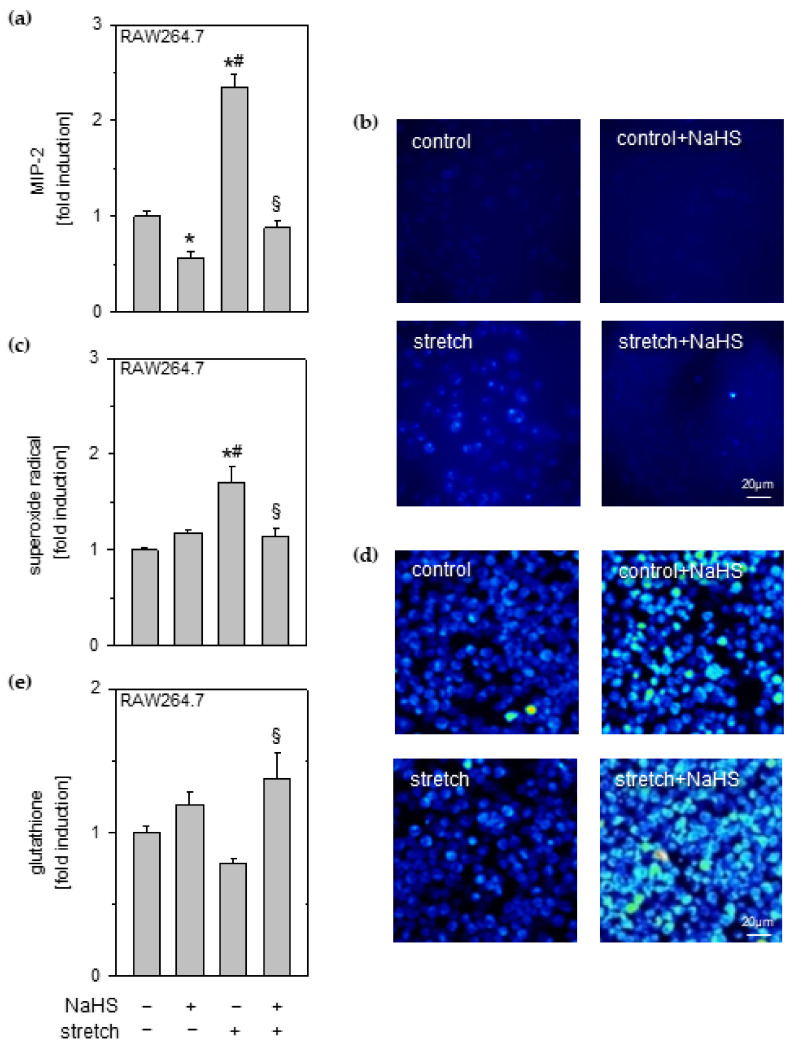
Effects of cyclic stretch and hydrogen sulfide on macrophages. RAW264.7 macrophage cells stretched and incubated with 1 mM NaHS, as indicated. (**a**) The amount of MIP-2 in cell medium supernatant was determined by ELISA. (**b**) Representative rainbow false-colored images of DHE staining for superoxide radicals and (**c**) the corresponding densitometric analysis. (**d**) Representative images of glutathione staining in macrophages and (**e**) the corresponding densitometric analysis. Data represent means ± SEM for n = 3/group. Analysis of variance (Tukey’s post hoc test), * *p* < 0.05 vs. control; # *p* < 0.05 vs. control + NaHS; § *p* < 0.05 vs. stretch.

In monoculture experiments, NaHS incubation reduced even baseline MIP-2 release in RAW264.7 macrophages as compared to untreated control cells (Figure 2a). In comparison to control conditions, mechanical stress profoundly increased MIP-2 release from macrophages that was prevented by additional NaHS administration (Figure 2a). 

Because pro-inflammatory signaling upon mechanical stress has been associated with oxidative stress in vivo [14], we next investigated superoxide radical formation. Superoxide radical formation (Figure 2b) in stretched macrophages was significantly increased as compared to unstretched cells (control and control + NaHS; Figure 2b,c). This was again prevented by NaHS supplementation (Figure 2b,c). 

Complementary to these findings, the antioxidant glutathione (Figure 2d) was slightly increased by NaHS in macrophages compared to control cells (Figure 2c,e). While stretch reduced glutathione formation, adding NaHS to stretched macrophages caused a significant increase in glutathione synthesis (Figure 2d,e).

### 3.4. Effects of Cyclic Stretch and NADPH Oxidase Inhibition on Reactive Oxygen Species and Inflammation on Macrophages

**Figure 3:**
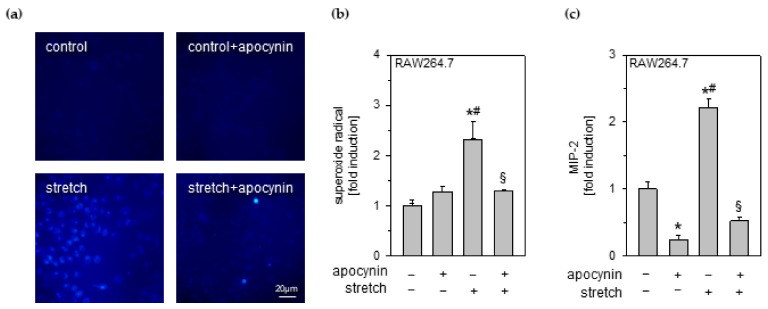
Effects of cyclic stretch and NADPH oxidase inhibition on ROS and inflammation on macrophages. RAW264.7 macrophage cells stretched and incubated with 2 mM apocynin as indicated. (**a**) Representative rainbow false-colored images of DHE staining for superoxide radicals and (**b**) the corresponding densitometric analysis. (**c**) The amount of MIP-2 in cell medium supernatant was determined by ELISA. Data represent means ± SEM for n = 3/group. Analysis of variance (Tukey’s post hoc test), * *p* < 0.05 vs. control; # *p* < 0.05 vs. control + apocynin; § *p* < 0.05 vs. stretch.

NADPH oxidases are one of the main sources of superoxide radicals [26]. We therefore applied the NADPH oxidase inhibitor apocynin to control and stretched macrophages and again determined the superoxide formation and inflammatory cytokine release in this context. Superoxide formation (Figure 3a) was comparable in the two control groups (control and control + apocynin). Cyclic stretch significantly increased the superoxide production in macrophages, which was prevented by apocynin incubation (Figure 3a,b). Likewise, MIP-2 release from stretched macrophages was increased compared to unstretched controls (Figure 3c), and again, apocynin completely prevented stretch induced MIP-2 release (Figure 3c).

### 3.5. Effects of Cyclic Stretch and Hydrogen Sulfide on Inflammation and Reactive Oxygen Species in Endothelial Cells

Besides epithelial cells and macrophages, endothelial cells also mediate early inflammatory signaling in response to cyclic stretch [27]. We therefore analyzed the effect of H_2_S on stretch-induced inflammatory and oxidative responses in HUVEC endothelial cells using GYY4137 as a slow H_2_S-releasing compound.

**Figure 4:**
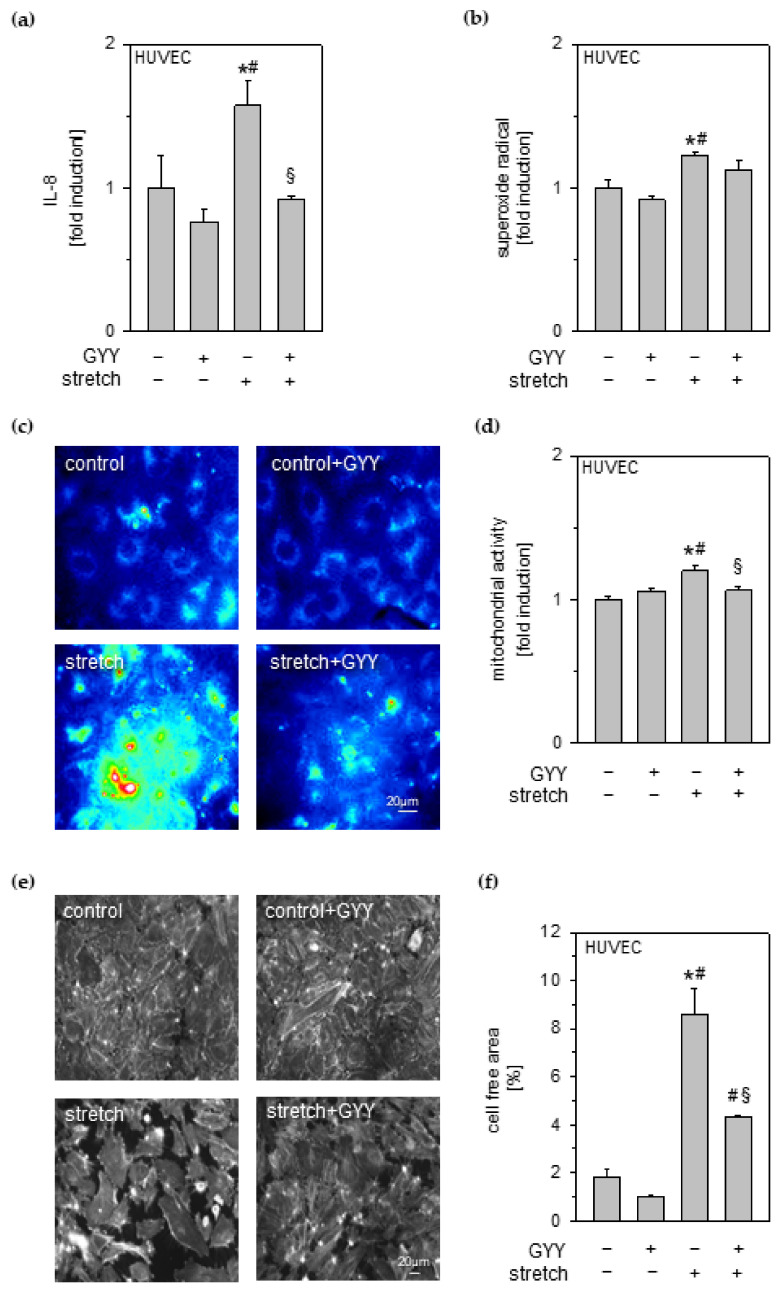
Effects of cyclic stretch and hydrogen sulfide on inflammation, ROS, and intercellular integrity in endothelial cells. HUVEC endothelial cells stretched and incubated with 1 mM GYY4137, as indicated. (**a**) The amount of IL-8 in cell medium supernatant was determined by ELISA. (**b**) Densitometric analysis of DHE staining for superoxide radicals. (**c**) Representative rainbow false-colored images of mitochondrial staining and (**d**) densitometric analysis of mitochondrial activity. (**e**) Representative images of actin fluorescence staining and (**f**) evaluation of cell free areas. Data represent means ± SEM for n = 3/group. Analysis of variance (Tukey’s post hoc test), * *p* < 0.05 vs. control; # *p* < 0.05 vs. control + GYY; § *p* < 0.05 vs. stretch.

Cyclic stretch induced a significant IL-8 release from HUVEC endothelial cells compared to unstretched control groups (Figure 4a). In contrast, H_2_S treatment prevented IL-8 release from HUVEC endothelial cells (Figure 4a).

Superoxide radical formation in endothelial cells was significantly increased due to cyclic stretch compared to both control groups (Figure 4b). Additional GYY4137 treatment partially decreased superoxide formation during stretch in endothelial cells (Figure 4b). 

MitoTracker stainings showed similar mitochondrial activity of unstretched controls without or with GYY4137 (Figure 4c). In contrast, cycling stretch induced mitochondrial activity in HUVEC cells (Figure 4c). Application of GYY4137 considerably reduced the intensity and size of active mitochondrial hotspots. Corresponding densitometry analysis likewise demonstrated a significant reduction of mitochondrial activity under cyclic stretch in endothelial cells when treated with GYY4137 (Figure 4d).

### 3.6. Effects of Cyclic Stretch and Hydrogen Sulfide on Endothelial Cell Integrity

Besides direct inflammatory and oxidative responses on a single cell level, disruption and dysfunction of the endothelial barrier during mechanical stress contributes to aggravation of inflammation and facilitates the influx of immune-competent cells such as neutrophils [28]. Therefore, we next evaluated whether H_2_S (GYY4137) would also modulate endothelial cell integrity during cyclic stretch.

Compared to the control groups (control and control + GYY), stretch disturbed the integrity of the HUVEC endothelial cell monolayer (Figure 4e), also depicted as the percentage of cell free area (Figure 4f). Additional GYY4137 treatment during cyclic stress in part preserved the endothelial integrity (Figure 4e,f). 

### 3.7. Effects of Hydrogen Sulfide and ROS Scavenging on Neutrophil Transmigration through Endothelial Monolayers

**Figure 5:**
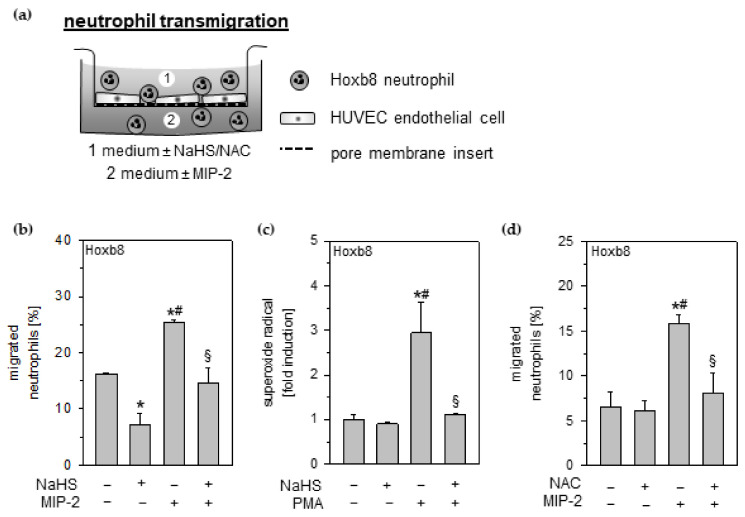
Effects of hydrogen sulfide and radical scavenging on neutrophil transmigration through the endothelial monolayer. (**a**) Co-culture setup of Hoxb8 neutrophil migration experiments. (**b**) Neutrophil migration was stimulated with 1.33 pg/mL MIP-2 in the presence of 1 mM NaHS, as indicated. The relative amount of migrated neutrophils was determined by flow cytometry. (**c**) Hoxb8 neutrophils were treated with 10 µM PMA and 1 mM NaHS, as indicated. ROS formation was detected by flow cytometry. (**d**) Neutrophil migration was stimulated with 1.33 pg/mL MIP-2 in the presence of 2 mM NAC, as indicated. The relative amount of migrated neutrophils was determined by flow cytometry. Data represent means ± SEM for n = 3/group. Analysis of variance (Tukey’s post hoc test), * *p* < 0.05 vs. control; # *p* < 0.05 vs. control + NaHS/control + NAC; § *p* < 0.05 vs. MIP-2/PMA.

The observed preservation of endothelial cell integrity upon H_2_S treatment may affect the ability of neutrophils to migrate through endothelial layers [13] toward chemoattractants, e.g., MIP-2. Thus, Hoxb8 neutrophils were added to a confluent unstretched monolayer of HUVEC endothelial cells seeded on a pore membrane (Figure 5a). In this co-culture setting, Hoxb8 neutrophils were able to migrate spontaneously through the endothelial monolayer. Additional NaHS incubation significantly reduced spontaneous neutrophil transmigration (Figure 5b). In the presence of MIP-2, the percentage of transmigrated neutrophils significantly increased, whereas MIP-2-induced transmigration was abolished in the presence of NaHS (Figure 5b).

Next, we examined ROS formation in neutrophils, its role in neutrophilic transmigration and finally its modulation capability by H_2_S and NAC. Compared to untreated or NaHS incubated controls, pro-oxidant phorbol 12-myristate 13-acetate (PMA) markedly increased ROS formation in Hoxb8 neutrophils. However, NaHS supplementation reduced ROS formation back to control levels despite the presence of PMA (Figure 5c). While incubation with NAC did not affect neutrophil migration within control groups, MIP-2-induced neutrophil transmigration through the endothelial barrier was completely abolished by NAC application (Figure 5d).

### 3.8. Effect of Hydrogen Sulfide on Neutrophil Chemotaxis

**Figure 6:**
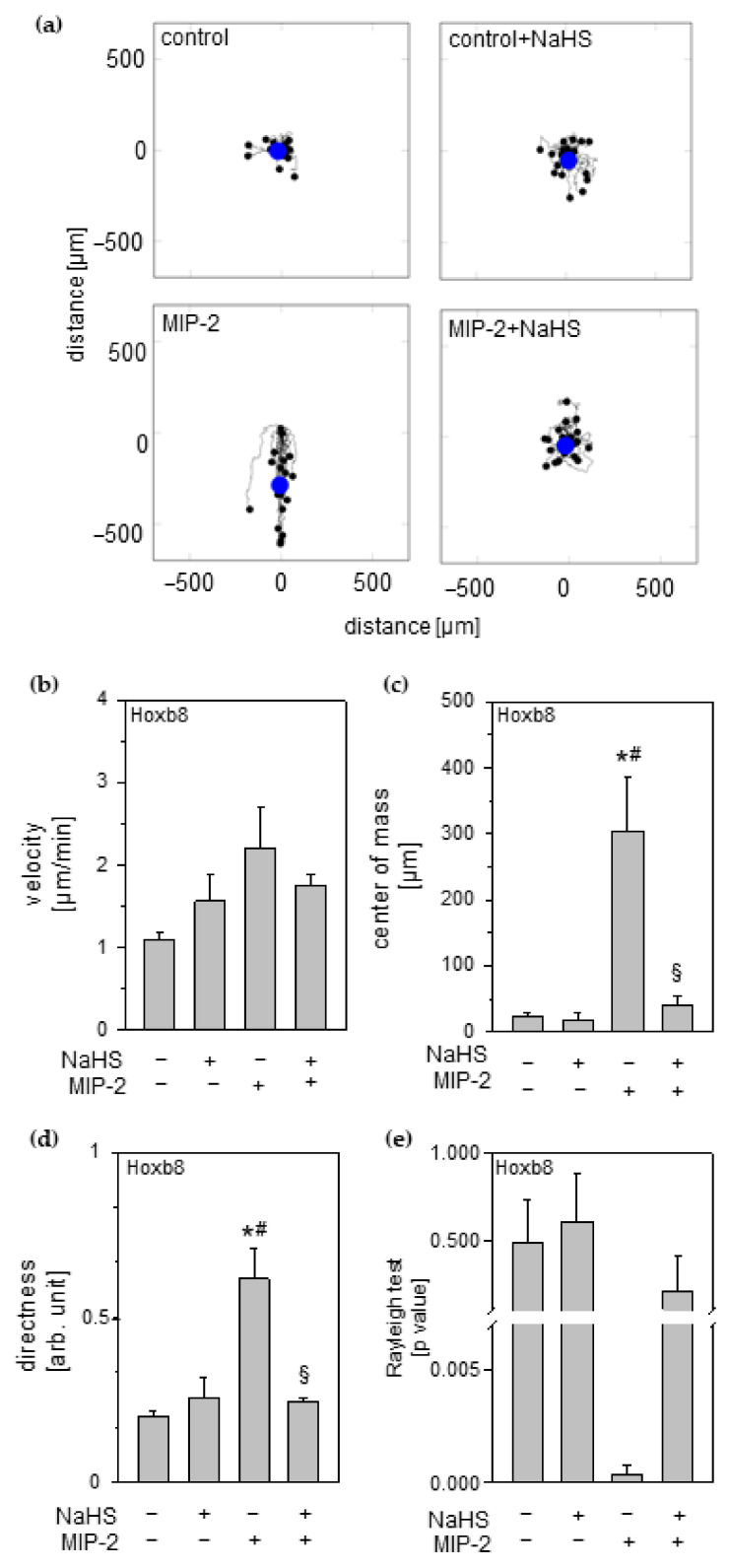
Effect of hydrogen sulfide on neutrophil chemotaxis Hoxb8 neutrophils were attracted with MIP-2 and treated with 1 mM NaHS, as indicated. The migration behavior of neutrophils was determined. (**a**) Representative trajectory plots: Starting points are set to zero and last tracking position marked with a black dot; the blue dot indicates the center of mass (average end position of tracked cells). Analysis of (**b**) velocity, (**c**) center of mass, (**d**) directness, and (**e**) Rayleigh test for the entire chemotaxis experiment. Data represent means ± SEM for n = 3/group. Analysis of variance (Tukey’s post hoc test), * *p* < 0.05 vs. control; # *p* < 0.05 vs. control + NaHS; § *p* < 0.05 vs. MIP-2.

We tested the direct effects of H_2_S on neutrophil migration capacity towards an inflammatory signal, e.g., MIP-2, in time-lapse imaging experiments. These experiments clearly revealed a distinct migration behavior of Hoxb8 neutrophils exposed to an MIP-2 gradient (Supplementary Video S1). Whereas neutrophils in unattracted control groups (control and control + NaHS) moved randomly around their primary positions, in chemotaxis groups, Hoxb8 neutrophils moved directly towards the MIP-2 gradient (MIP-2). This straight orientation was lost after NaHS treatment, also mirrored in the corresponding trajectory plots (Figure 6a). Additional evaluation of relevant chemotaxis parameters from these experiments revealed the following: Compared to controls, MIP-2 incubation of Hoxb8 showed: (1) a slight increase in the speed of movement (velocity; Figure 6b); (2) significantly different movement pattern (center of mass; Figure 6c); (3) a significantly higher straightness of migration (directness; Figure 6d), the latter also confirmed in (4) Raleigh’s test for directed cell migration (*p* = 0.004, Figure 6e). By contrast, application of NaHS to the MIP-2 gradient eliminated all of the above-described chemotactic effects, i.e., center of mass, directness, and Rayleigh *p*-value were comparable to controls despite chemotactic stimulation (Figure 6b–e).

## 4. Discussion

While H_2_S has been shown to prevent lung injury in vivo, it still remains completely unclear which pulmonary cell types and what kind of cellular cross talk between them mediate these protective effects. Therefore, we aimed to systematically analyze how H_2_S affects the most important pulmonary cell types involved in the onset and propagation of mechanical-stress-induced responses in the current study.

As demonstrated here and as shown previously, alveolar A549 und bronchiolar BEAS-2B epithelial cells reacted with the release of pro-inflammatory cytokines on mechanical stress [8,9]. However, our findings show that H_2_S (NaHS) does not interfere with the onset of inflammation in A549 or BEAS-2B cells–even though we extensively tested H_2_S on stretched A549 cells in a wide NaHS concentration range (Supplementary Figure S1). These results are surprising, because anti-inflammatory effects of H_2_S on epithelial cells were described recently, e.g., inhibition of LDH release in LPS-challenged murine epithelial cells [29]. To further examine the conflicting results and to study whether the lack of anti-inflammatory effects of NaHS on epithelial cells might be limited to mechanical stretch insults, we induced the pro-inflammatory cytokine release by LPS or TNF-α stimulation. Similarly, LPS-/TNF α –induced cytokine release proved to be unaffected by NaHS (Supplementary Figure S2). In the light of these findings, it is plausible to speculate that H_2_S-mediated anti-inflammatory effects may probably not only depend on the challenge applied, but also on the epithelial cell type, the species (mice vs. human), the way of immortalization (SV40 transformed vs. adenocarcinoma), the pharmacokinetics of the H_2_S-releasing compound [20], or a combination of all of these factors.

Besides epithelial cells, macrophages as cells of the innate immune system mediate mechanically induced inflammatory responses [30]. In fact, our results from co-culture experiments, mimicking a crosstalk between stretched epithelial and alveolar macrophages in the lung, demonstrate that unstretched macrophages release pro-inflammatory cytokines as a response to epithelial cell stretching. These findings are supported by previous reports, describing paracrine cell communication as sufficient to activate macrophages during the progression of inflammation [31,32]. We further demonstrate that H_2_S treatment prevents MIP-2 release from macrophages, suggesting that H_2_S can inhibit the propagation of inflammation that is triggered by paracrine signaling. Since models of LPS- [33,34] or free fatty acids-stimulated macrophages [35] also showed reduced pro-inflammatory cytokine formation after H_2_S treatment, we assume that H_2_S may act as an anti-inflammatory, specifically in macrophages.

Macrophages are not limited to promoting stretch signals released by epithelial cells. Former reports showed that murine and human macrophages respond to mechanical stress with the release of pro-inflammatory cytokines [10,30]. As confirmed in the current study, H_2_S prevented MIP-2 release from stretched macrophages [10]. Although the role of hydrogen sulphide signaling in macrophages is well studied [30,36], not all underlying molecular mechanisms are known. 

In this respect, it has been demonstrated that stretched macrophages respond to mechanical stress with ROS production [37], and H_2_S may affect ROS signaling in vivo and in vitro [22,23,38]. It is commonly accepted that the induction of ROS, and particularly superoxide radicals, initiates the upregulation of inflammatory cytokines [39]. We therefore assessed whether ROS/H_2_S may regulate this mechanical stress-induced inflammatory response. As confirmed by our results, H_2_S largely prevented superoxide radical production in stretched macrophages while simultaneously maintaining glutathione levels. 

The key source of superoxide radical formation under mechanical stress is represented by the enzymatic activity of NADPH oxidases [40,41,42]. We therefore inhibited NADPH activity in stretched macrophages by apocynin and clearly show that this intervention abolishes stretch-induced ROS formation. In this respect, it is important to note that apocynin itself has anti-inflammatory effects [43,44]. Nevertheless, previous studies have shown that blocking the superoxide radical formation suppresses MIP-2 release, supporting the assumption that ROS-signaling is required for a pro-inflammatory cytokine response [23,40]. Altogether, we suggest that the anti-oxidative properties of H_2_S, including radical scavenging (e.g., via glutathione) and/or regulation of ROS synthesis (e.g., via NADPH oxidases) [23,45,46,47] represent a key mechanism in H_2_S-mediated modulation of inflammatory signaling in mechanically stressed macrophages. 

Besides epithelial cells and macrophages, endothelial cells also mediate early inflammatory signaling in response to stretch [27]. As presented here and demonstrated earlier [48,49], HUVEC endothelial cells respond with pro-inflammatory cytokine release to cyclic stretch. In our study, supplementation with H_2_S prevented stretch-induced inflammatory response in endothelial cells. Data on the effects of H_2_S and cyclic stretching on endothelial cells are missing. However, in other models, H_2_S has been shown to prevent the onset of inflammation in DL-propargylglycine- [50], angiotensin II-stimulated [51] or theophylline-stimulated endothelial cells accompanied by limited ROS formation [38,51]. ROS formation in endothelial cells, e.g., induced by mechanical load, is promoted by activation of mitochondria [52], and H_2_S may prevent ROS in endothelial cells through preservation of the mitochondrial activity [53]. Likewise, our results clearly show that stretch increases mitochondrial activity or count, which is inhibited in the presence of H_2_S, thus strongly suggesting that H_2_S downregulates ROS synthesis in cyclic stretched endothelial cells by modulating its mitochondrial activity.

As a result of unphysiological stretching evoking inflammatory and oxidative responses within the lungs, the endothelial barrier function may also be severely disturbed. The latter would itself aggravate inflammatory and oxidative injuries [27,48,54]. Our results demonstrate that H_2_S conserves the endothelial intercellular integrity despite stretching that, in the absence of H_2_S, disrupts the cell barrier. Another aspect of stretch-impaired endothelial cell integrity is the facilitated transmigration of neutrophilic cells [28]. Transmigration of activated neutrophils displays a critical step in the development of VILI [13]. Therefore, preservation of the endothelial barrier function may be an important mechanism, limiting neutrophil transmigration in the lung [55,56]. Our results as well as previous reports clearly demonstrate that neutrophil transmigration through the endothelial barrier is significantly enhanced in a pro-inflammatory environment [13,57]. In contrast, the presence of H_2_S abolishes neutrophil transmigration upon pro-inflammatory stimuli in our study.

In conjunction with the above-described effects on endothelial cells, our findings indicate that H_2_S may, on the one hand, inhibit neutrophil transmigration by preservation of the endothelial barrier. On the other hand, the presence of H_2_S significantly reduced neutrophil transmigration even in the absence of chemoattractant, supporting the notion that H_2_S may directly affect neutrophil migrational behavior in addition to preserving the endothelial barrier function. The latter suggestion can be underscored by previous studies, showing H_2_S as an effector on neutrophil rolling and adhesion on endothelial cells, thus modulating receptors at both cell sites [19,58].

Finally, superoxide radical signaling has been demonstrated to be another main regulator of neutrophil transmigration and chemotaxis [59]. As proved by the current and a previous study, Hoxb8 neutrophils can rapidly release superoxide radicals upon PMA or LPS stimulation [57]. Conversely, H_2_S treatment clearly prevented superoxide radical formation in neutrophils under mechanical stress in this study. These findings subsequently underline the possibility that H_2_S may limit neutrophil migration by preventing ROS signaling in neutrophils. Indeed, as demonstrated in the current study, ROS reduction by another radical scavenger, i.e., NAC, also prevents neutrophil migration, again supporting the idea that ROS signaling acts as a key mechanism involved in H_2_S-mediated inhibition of neutrophil transmigration. 

The setting and conduction of the above-described experiments could not exclude potential direct effects of H_2_S on neutrophils and their movement. In live imaging chemotaxis experiments, Hoxb8 neutrophils moved straight towards the chemoattractant signal, thereby emphasizing results from a recent study in human neutrophils [60]. Most interestingly, Hoxb8 neutrophils completely lost their straight movement towards MIP-2 in the presence of H_2_S. Taking into account that ROS are essential for neutrophil orientation [59] and having proved in the current study that radical scavenging likewise prevents neutrophil transmigration, we suggest that by reducing superoxide radical formation, H_2_S leads to a ‘disorientation’ of neutrophilic cell movements, thus revealing a novel mechanism that contributes to reduced neutrophil chemotaxis and protection.

## 5. Conclusions

**Figure 7:**
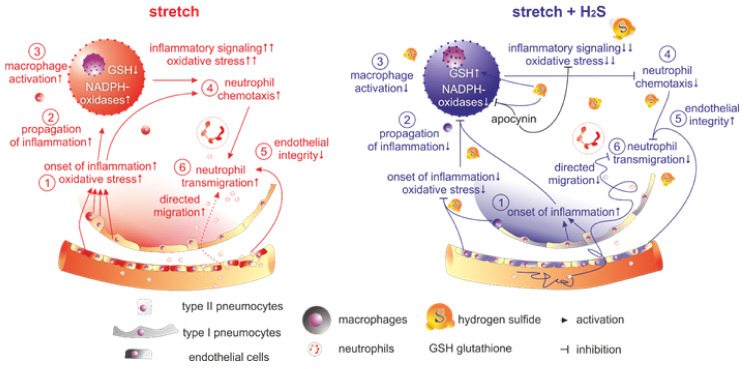
Summary of the effects of cyclic stretch and H_2_S on onset and propagation of inflammation. S. Serial numbers indicate onset and propagation of signaling.

Altogether, cell stretch and co-culture experiments are suitable setups to explore the effects of H_2_S-releasing compounds on the onset and propagation of inflammation and provide explanations for H_2_S-mediated protective mechanisms. Our findings suggest that H_2_S orchestrates a series of cell-specific inflammatory responses to mechanical stress. As summarized in Figure 7, H_2_S (NaHS) does not affect the onset of inflammatory signaling in stretched epithelial cells (A549 and BEAS-2B) but prevents the propagation of inflammation due to paracrine crosstalk between epithelial cells and macrophages (RAW264.7). H_2_S completely abolishes stretch-induced MIP-2 release in macrophages, apparently by inhibiting excessive ROS formation. NADPH oxidases appear to play an important role in transduction of mechanical stress into inflammatory signaling in macrophages. Moreover, reduced oxidative stress in the presence of H_2_S evidently contributes to the decrease of pro-inflammatory cytokine release, oxidative stress, and preservation of endothelial integrity in stretched HUVEC cells. Finally, H_2_S directly distracts neutrophil chemotaxis and transmigration by a mechanism involving control of ROS steady state.

## Figures and Tables

**Table 1:** Cell stretch experiments–experimental settings. Instrument setup and experimental conditions were optimized prior to the experiments for each cell type. V = volume, Supp = supplements, C = concentration, − = without supplements, + = with supplements.

Cell Line	Plate Coating	Medium		H_2_S		Elongation		
		Volume	Supplements	Source	Conc.	Shape	[%]	[h]
A549	Collagen IV	1 mL	-	NaHS	1 mM	Square	20.5	4
BEAS-2B	Collagen I	1 mL	+	NaHS	1 mM	Square	20.5	6
RAW264.7	Collagen I	1 mL	-	NaHS	2 mM	½ Sinus	20.5	6
HUVEC	Collagen IV	2 mL	+	GYY	1 mM	Sinus	20.0	24

## Data Availability

The data that support the findings of this study are contained in the article.

## Supplementary Materials

The following supporting information can be downloaded at: https://www.mdpi.com/article/10.3390/antiox11051001/s1, Figure S1: Effect of H_2_S in various concentrations and strain on inflammatory response in A549 epithelial cells; Figure S2: Effect of H_2_S and LPS or TNF-α on inflammatory response in A549 epithelial cells; Video S1: Effect of H_2_S on neutrophil migration.
