# Supplementary material for: Profiling Distinctive Inflammatory and Redox Responses to Hydrogen Sulfide in Stretched and Stimulated Lung Cells
Source: Antioxidants (Basel). 2022 May 19;11(5):1001. doi: 10.3390/antiox11051001 (PMC9137934; doi:10.3390/antiox11051001)
Supplement: Supplementary file 1 [file antioxidants-11-01001-s001.zip › Spassov et al._Supplementary Materials.pdf]

Supplementary materials

Supplementary video S1

Effect of H_2S on neutrophil migration

Neutrophil migration was assessed in ibidi µ-slides chemotaxis tool. The observation channel and surrounding chambers were filled with growing medium (control) or medium containing NaHS (control+NaHS). A chemotaxis gradient was created by adding MIP-2 to one of the µ-slide chambers in the absence (MIP-2) or presence of NaHS (MIP-2+NaHS). Images captured every 2.5 min for 4 hours were imported as sequence in ImageJ software and saved as a video with a 7 frames/s.

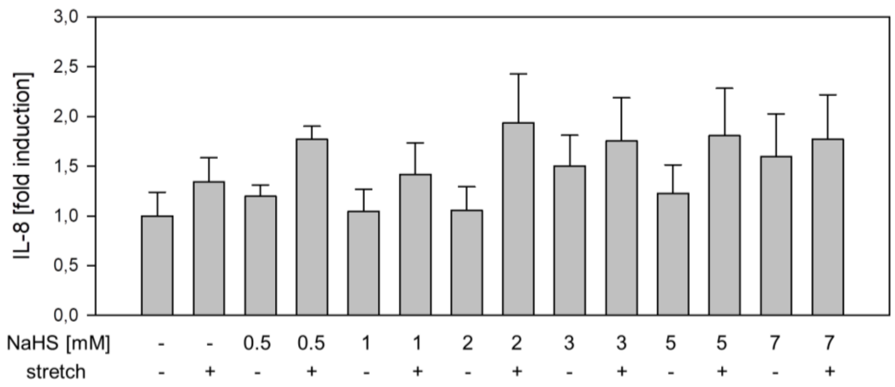

Supplementary Figure S1. Effect of H_2S in various concentrations and strain on inflammatory response in A549 epithelial cells

A549 epithelial cells were stretched and incubated for 4 h with 0.5 to 7 mM NaHS as indicated. The amount of IL-8 in cell medium supernatant was determined by ELISA. Data represent means ± SEM for n=3/group. Analysis of variance.

The results demonstrated no effect of NaHS (in the investigated range) on the inflammatory response in stretched A549 epithelial cells.

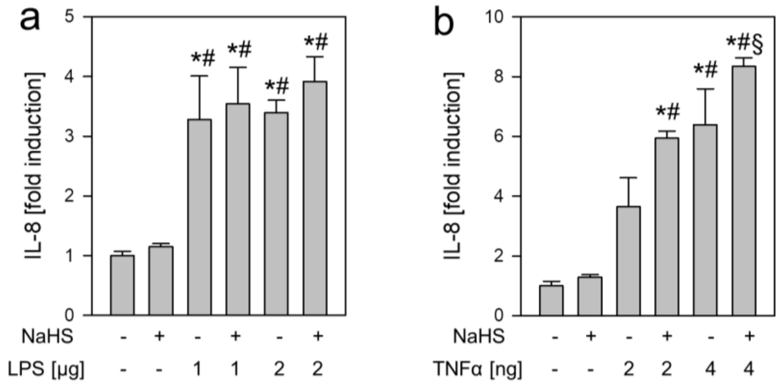

Supplementary Figure S2. Effect of H_2S and LPS or TNFα on inflammatory response in A549 epithelial cells

A549 epithelial cells were incubated with varying concentrations of (a) LPS (E.coli 055:B5, Sigma, Taufkirchen, Germany) for 8 h or with (b) TNFα (Sigma) for 4 h as indicated. The amount of IL-8 in cell medium supernatant was determined by ELISA. Data represent means ± SEM for n=3/group. Analysis of variance (Tukey`s post hoc test), $^{*}P<0.05$ vs. control; $^{\#}P<0.05$ vs. control+NaHS; $^{\S}P<0.05$ vs. 2 ng TNFα.

The results demonstrated no effect of NaHS on the inflammatory response in LPS or TNFα stimulated A549 epithelial cells.
